# Altered Domain Functional Network Connectivity Strength and Randomness in Schizophrenia

**DOI:** 10.3389/fpsyt.2019.00499

**Published:** 2019-07-23

**Authors:** Victor M. Vergara, Eswar Damaraju, Jessica A. Turner, Godfrey Pearlson, Aysenil Belger, Daniel H. Mathalon, Steven G. Potkin, Adrian Preda, Jatin G. Vaidya, Theo G. M. van Erp, Sarah McEwen, Vince D. Calhoun

**Affiliations:** ^1^Tri-institutional Center for Translational Research in Neuroimaging and Data Science (TReNDS), Georgia State University, Georgia Institute of Technology, Emory University, Atlanta, GA, United States; 2The Mind Research Network, Albuquerque, NM, United States; ^3^Psychology Department Georgia State University, Atlanta, GA, United States; ^4^Neuroscience Institute, Georgia State University, Atlanta, GA, United States; ^5^Departments of Psychiatry and Neuroscience, Yale University School of Medicine, New Haven, CT, United States; ^6^Olin Neuropsychiatry Research Center, Institute of Living, HHC, Hartford, CT, United States; ^7^Department of Psychiatry, University of North Carolina, Chapel Hill, NC, United States; ^8^Department of Psychiatry and Weill Institute for Neurosciences, University of California, San Francisco, San Francisco, CA, United States; ^9^Mental Health Service, Veterans Affairs San Francisco Healthcare System, San Francisco, CA, United States; ^10^Department of Psychiatry and Human Behavior, University of California, Irvine, Irvine, CA, United States; ^11^Department of Psychiatry, University of Iowa, IA, United States; ^12^Translational Neuroscience Laboratory, Department of Psychiatry and Human Behavior, University of California, Irvine, Irvine, CA, United States; ^13^Center for the Neurobiology of Learning and Memory, University of California, Irvine, Irvine, CA, United States; ^14^Pacific Neuroscience Institute, Santa Monica, CA, United States

**Keywords:** functional MRI, functional network connectivity, randomness, schizophrenia, connectivity strength function

## Abstract

Functional connectivity is one of the most widely used tools for investigating brain changes due to schizophrenia. Previous studies have identified abnormal functional connectivity in schizophrenia patients at the resting state brain network level. This study tests the existence of functional connectivity effects at whole brain and domain levels. Domain level refers to the integration of data from several brain networks grouped by their functional relationship. Data integration provides more consistent and accurate information compared to an individual brain network. This work considers two domain level measures: functional connectivity strength and randomness. The first measure is simply an average of connectivities within the domain. The second measure assesses the unpredictability and lack of pattern of functional connectivity within the domain. Domains with less random connectivity have higher chance of exhibiting a biologically meaningful connectivity pattern. Consistent with prior observations, individuals with schizophrenia showed aberrant domain connectivity strength between subcortical, cerebellar, and sensorial brain areas. Compared to healthy volunteers, functional connectivity between cognitive and default mode domains showed less randomness, while connectivity between default mode-sensorial areas showed more randomness in schizophrenia patients. These differences in connectivity patterns suggest deleterious rewiring trade-offs among important brain networks.

## Introduction

The disconnection hypothesis (1) is an important landmark in understanding the underpinnings of schizophrenia. It proposed that the brain disconnections in schizophrenia are more of a functional nature rather than anatomical. Later studies provided validation for the existence of disconnections in the brain of schizophrenia patients (2–4). Functional connectivity studies using resting state data have provided important insights into aberrant functional connectivity patterns of specific brain areas/networks in schizophrenia compared to healthy controls (5). Using a different perspective, aberrations may not necessarily be singled out in one brain region or network but may affect patterns of connectivity that involve the whole brain. Graph measures have been an important tool in revealing aberrant patterns of functional connectivity involving nodes distributed throughout the brain (6, 7). However, more research is needed to relate abnormalities occurring in small specific brain areas/networks with those observed in whole brain analyses.

**Figure 1:**
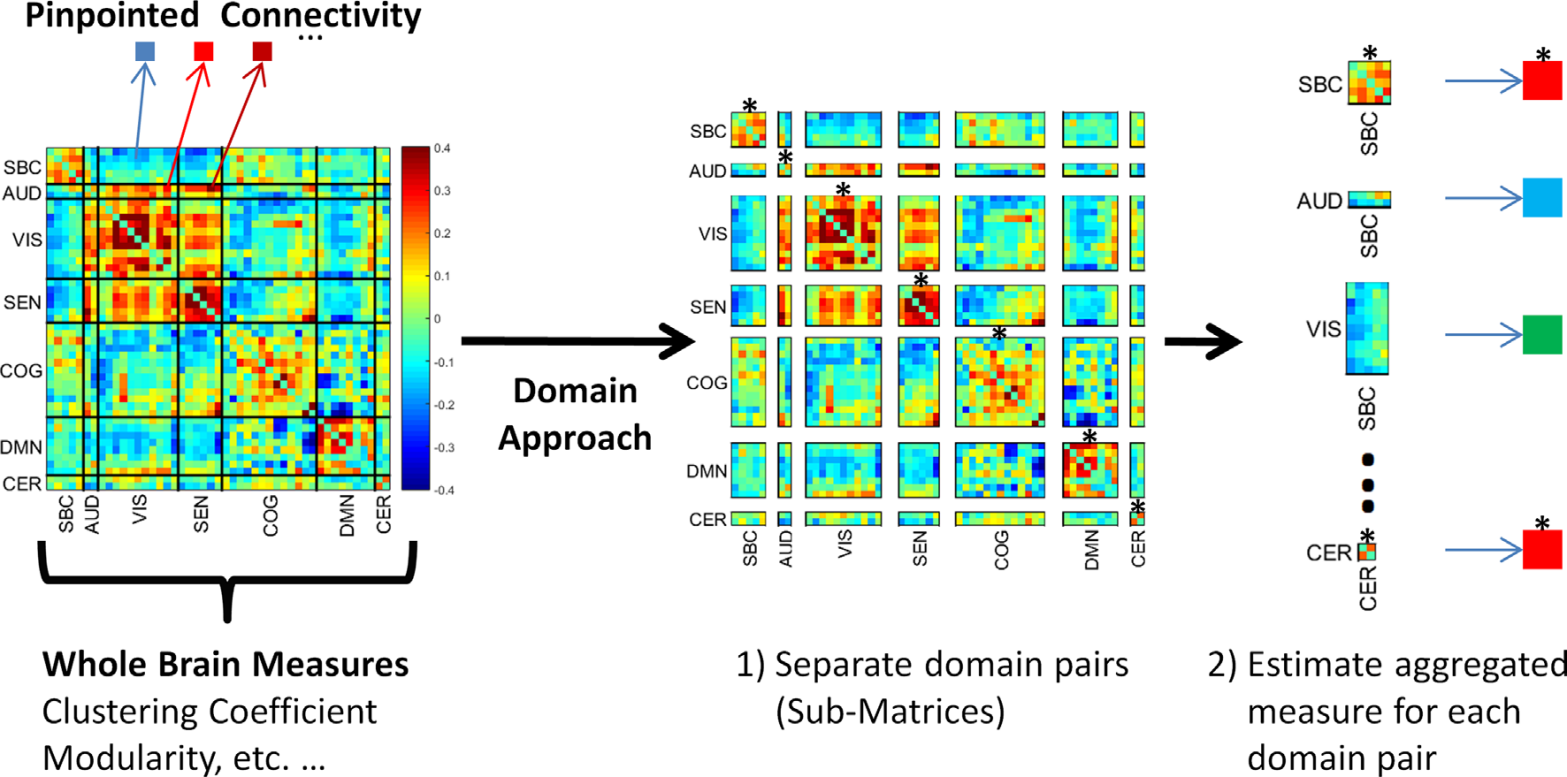
The domain functional connectivity approach. Instead of estimating whole brain measures or considering single correlations, the domain approach works with the submatrices of the functional network connectivity matrix. The first step 1) is to separate functional connectivity domain submatrices. The second step 2) is to aggregate the values using a meaningful measure. The figure shows within domain connectivity indicated by an asterisk on top of the submatrices. Notice this set represents connectivity of a domain with itself. Asterisk-marked submatrices are SBC-SBC, AUD-AUD, VIS-VIS, SEN-SEN, COG-COG, DMN-DMN, and CER-CER. These submatrices are located in the main diagonal of the whole brain matrix.

Our group has recently refocused attention from single brain networks to groupings of brain networks also called domains (8–10). This change in focus is achieved through functional network connectivity (FNC) analyses in which spatio-temporal properties of brain resting state networks (RSNs) are estimated for further analysis (11). Nominal FNC analysis assesses the relationships between two different RSNs. Previous studies have found that schizophrenia affects the FNC of many RSN pairs providing details for very specific and localized brain areas (5). Yet, results from that work suggest that many areas of the brain are similarly affected by schizophrenia in spite of being independently analyzed. For example, **Figure 2** in Ref. (5) shows how the bulk of independent results concentrate in areas such as the visual and sensorimotor domains with consistent direction of effects. This observation suggests that schizophrenia abnormalities might affect in a similar way all RSNs within a domain and opens the possibility of studying the domain as a group of RSNs with common effects. Our current work follows by using methods that can fuse information from several RSNs allowing for a stronger RSN group effect. The basic functional domain approach considers two subsets of RSNs from a pair of functional domains (see **Figure 1**). Information from all RSNs within the domains is then fused to obtain a domain-based assessment. The analysis is then performed on all available pairs of domains. This approach studies the brain at a middle point between coarser whole brain and finer per-RSN analysis. Domain analysis has revealed specifics of information exchange among domains with strong effects related to schizophrenia on audio-visual and sensorimotor (AVSN) domains (8). However, FNC in schizophrenia has not been analyzed using a domain-focused approach.

In this work, we investigated domain FNC (see **Figure 1**) differences between individuals with schizophrenia and healthy volunteers. Two different domain FNC measures are examined: 1) domain connectivity strength, and 2) randomness. Domain connectivity strength is the average of all connectivity values linking two domains. In **Figure 1**, this is equivalent to submatrix averaging of connectivity strengths between each domain pair. Previously, connectivity strength has been used to describe whole brain averages as a way of obtaining a single aggregated connectivity value for each brain (12). Domain-wise connectivity strength is expected to somewhat resemble results previously obtained with single connectivity values (5). We have also employed the same domains from this previous study because the RSN grouping was algorithmically processed to find the optimal set of functional domains. Another important concept for the research community is the existence of patterns characterizing functional brain connectivity. The application of graph theoretical measures to whole brain connectivity matrices has revealed the presence of such patterns in the functional connectivity matrix (13) as well as aberrant patterns in schizophrenia (14). However, known graph theoretical measures, such as modularity, are not suitable for functional domain connectivity. The reason is the existence of many rectangular and non-symmetric connectivity submatrices that would not fit the assumptions of a symmetric and square matrix used in estimating graph theoretical measures (see **Figure 1**). We employ a different concept known as randomness to measure differences in the structure of functional connectivity within domain submatrices. The basic idea is to estimate the degree of difference between a submatrix of interest and a random submatrix.

The word randomness can be conceptualized as the absence of predictability. A single valued random variable is undetermined and can then assume any value. As corollary, lack of predictability generally includes the lack of a recognizable pattern. However, the Central Limit Theorem does present us with a pattern of a bell shape curve as the number of included variables increases. Describing random or unexplained variability of FNC assessments presents additional complications because they might not be identically distributed (may exhibit different means and variances) and might not be independent variables. In practice FNC data are best represented in matrix form instead of the single variable representation; thus, our study of FNC randomness employs the Random Matrix Theory to best characterize the FNC data. A random matrix can be described as a matrix array of random variables (15). Related to the Central Limit Theorem, Random Matrix Theory focuses on the pattern of eigenvalues which in square matrices is described by the Circular Law (16). To apply the theory for square and rectangular matrices, it is preferable to use the singular value (SV) spectrum which has its own predictable pattern (17). Randomness is then assessed through the similarity between the typical random SV spectrum and the SV spectrum of the matrix of interest. The randomness measure (denoted by *L*) has been defined as the Mahalanobis distance between the two SV spectra (10). In this randomness measure, large *L* values suggest the presence of non-random matrix patterns. Hypothesis testing can be performed because *L* follows a chi square distribution, after appropriate normalization, and its *p* value can be determined. We examined randomness at the domain connectivity level seeking for differences between individuals with schizophrenia and controls. The subject group with lower *L* value will then present more random domain connectivity with and less structure of its domain matrix representation.

Outcomes for domain-wise randomness are difficult to anticipate since this is the first time such analysis is performed. The first clue can be found by considering that graph measures are sensitive to changes in brain connectivity structure in a similar way that randomness is sensitive to structure unpredictability. Previous work using graph theoretical measures and a smaller sample (19 schizophrenia and 19 controls) suggests a set of areas susceptible to connectivity structure changes including frontal, parietal, occipital, and cerebellar regions (6). Several studies based on diffusion tensor imaging indicated abnormalities in white matter tracts (18, 19) that might result in a generalized more random connectivity in schizophrenia. There is also evidence of a hyperactive default mode network in schizophrenia patients (20) as well as changes in the spatial location of the network (21) suggesting an altered architecture that might be detected by the randomness measure. We believe that randomness will provide further evidence for the existence of abnormal domain connectivity patterns in schizophrenia that might help understand this neurodegenerative disease.

## Materials and Methods

### Subjects

This study employed a BOLD fMRI data set from 163 healthy controls (HC) along with 151 schizophrenia patients (SZ) with similar mean age and gender distribution, collected from seven different institutions in the United States (5). The HC group consisted of 117 males and 46 females (mean age 36.9). For statistical analyses, we selected the subset of 119 SZ patients for which medication data were available in the form of chlorpromazine equivalence scores (CPZ) (5). The final SZ group consisted of 90 males and 29 females (mean age 37.7). Prior to participation, informed consent was obtained from each subject in compliance with the Internal Review Boards of corresponding institutions. A neurocognitive profile was obtained from all participants using the computerized multiphasic interactive neurocognitive system (CMINDS) (22) composed of six domains: speed of processing, attention/vigilance, working memory, verbal learning, visual learning, and reasoning/problem solving. The procedure generally takes less than 1 h and 30 min to complete. The CMINDS was developed more than a decade ago and is well validated against both the MATRICS battery and the ADASCog (23, 24). Positive and negative syndrome scale (PANSS) values were also collected from each SZ subject (for sample details see **Supplementary Table 1**).

### Data Collection and Preprocessing

Data from six of the seven sites were collected using a 3T Siemens TIM Trio System. A 3T General Electric Discovery MR750 scanner was used at the remaining site. Resting state fMRI scans were acquired using a standard gradient-echo echo planar imaging paradigm: FOV of 220 × 220 mm (64 × 64 matrix), TR = 2 s, TE = 30 ms, FA = 77^0^, 162 volumes, 32 sequential ascending axial slices of 4 mm thickness and 1 mm skip. Subjects had their eyes closed during the resting state scan.

Data processing was performed using a combination of toolboxes (AFNI, SPM, and GIFT) and custom code written in Matlab. We performed rigid body motion correction using the INRIAlign (25) toolbox in SPM to correct for subject head motion followed by slice-timing correction. Next, the fMRI data were despiked using AFNI’s 3dDespike algorithm to mitigate the impact of intensity outliers. The fMRI data were subsequently warped to a Montreal Neurological Institute (MNI) template and resampled to 3 mm^3^ isotropic voxels. Instead of Gaussian smoothing, we smoothed the data to 6 mm full width at half maximum (FWHM) using AFNI’s BlurToFWHM algorithm which performs smoothing by a conservative finite difference approximation to the diffusion equation. This approach has been shown to reduce scanner-specific variability in smoothness providing “smoothness equivalence” to data across sites (26). Each voxel time course was variance normalized prior to performing group independent component analysis as this has shown to better decompose subcortical sources in addition to cortical networks.

We employed group independent component analysis (gICA) as implemented in the GIFT Toolbox (http://mialab.mrn.org/software/gift/) to obtain a set of maximally independent RSNs (27, 28). The gICA in the GIFT toolbox is designed to optimize spatial independence thus optimizing spatial segregation. Spatial and temporal information were collected as the outcome of the GIFT Toolbox. FNC was computed as the pairwise correlation between RSN time courses. Time courses were band pass filtered using a [0.01–0.15] Hz fifth-order Butterworth filter prior to computing FNC. The mean FNC matrix was organized into modular partitions, each partition corresponding to a functional domain, using the Louvain algorithm of the brain connectivity toolbox (https://sites.google.com/site/bctnet/). This RSN grouping has been utilized many times before in schizophrenia literature being one of the main reasons to pick this configuration (5, 29–31). The algorithmic originally obtained using algorithmic methods underwent human inspection by subject matter experts. The functional domains in the FNC matrix are depicted in **Supplementary Figure 1** and include sub-cortical (SBC) domain, auditory (AUD) domain, visual (VIS) domain, sensorimotor (SEN) domain, a broad set of regions involved in cognitive control and attention (COG), default-mode network (DMN) regions, and cerebellum (CER). Spatial maps for this set of domains can be found in Ref. (5). We will adopt the previously defined set of functional domains in our work.

### Connectivity Strength

The set of FNC values, obtained from correlating time courses, constitute a detailed description of brain connectivity. These FNC values represent the relationships between pairs of spatially localized brain areas but do not provide a whole brain level summary. We previously reported on connectivity abnormalities in schizophrenia after analyzing FNC data (5). In the current work, we emphasize on analyzing groups of FNCs. Two different levels of grouping are considered: whole brain and domain level. The whole brain level is based on assessing the whole brain connectivity strength which is computed by averaging all FNC values into one single value per subject (12, 14). We use this connectivity strength to study whole brain effects. The next step is to restate the connectivity strength concept following a functional domain focus previously proposed by our group (8, 9). The domain level consists of assessing domain connectivity strength as the average correlation over all RSN pairs belonging to one or two domains. Within domain connectivity occurs when two domains are the same, i.e., the set of FNCs involves RSNs within the same domain. Between domains connectivity occurs when the FNCs are based on correlations involving RSNs from two different domains. This way, we considered all 28 pairs of domains [SBC-SBC, SBC-AUD, SBC-VIS, SBC-SEN, …, CER-CER]. If arranged as a matrix, the FNC values from a specific domain pair forms a submatrix of the whole brain matrix. **Figure 1** displays the partition of the whole brain matrix into within and between domain submatrices. All within domain submatrices are square (same number of rows and columns) and symmetric (a symmetric matrix is equal to its transpose). Between domain submatrices can be square or rectangular (the number of rows might not equal the number of columns), but they are all non-symmetric. Irrespective of its size, values within submatrices are averaged to estimate the domain connectivity strength of the corresponding submatrix. A set of 28 different domain connectivity strength values is estimated for each subject.

We tested the relationships between connectivity strength and CMINDS scores using linear regression models. The set of tests included separate models for whole brain and domain grouping levels. Multicomparison correction was performed using the false discovery rate (FDR) method. Each model included age, sex, mean frame-wise displacement (meanFD), CPZ, and collection site as confounding factors of no interest. The meanFD regressor was included as a measure of head movement, and it is obtained by averaging the backwards difference of realignment estimates from each scan (32). Since diagnosis was found to influence CMINDS results, we repeated the tests including diagnosis and interaction terms along with age, sex, meanFD, CPZ, and site. In addition, linear regressions examined relationships between connectivity strength and schizophrenia symptom severity measures, including general, negative, and positive PANSS scores. These analyses included the same confounding variables.

We also performed group test analyses for possible significant differences between HC and SZ groups. For the purpose of group tests only, all connectivity strength values were first orthogonalized with respect to the confounding factors CPZ, age, sex, meanFD, and collection site. We included meanFD (33) to correct for individual differences in residual motion following suggestions in previous publications (34, 35). Finally, two sample *t*-tests comparing connectivity strength between groups was performed at the whole brain and at the domain levels. Multiple comparison corrections were performed using the FDR method.

### Graph Modularity

Graph measures are derived from adjacency matrices representing the brain connectivity organization (36, 37). However, two characteristics of adjacency matrices are that they exhibit nodal symmetry and are composed of weights (with a range between 0 and 1). A matrix exhibits node symmetry if two edge values are defined for every pair of different nodes. If the two edge values are equal, the matrix is symmetric; otherwise the matrix is not symmetric. Notice that node symmetry requires the matrix to be square, but this condition does not comply with most domain connectivity submatrices. The main reason is that domain analysis may result in rectangular submatrices without node symmetry as illustrated in **Figure 1**. For this reason, graph measures are not applied to domain connectivity in this work. We will focus on whole brain FNC matrices which are symmetric, and methods to estimate the adjacency matrix from FNC matrices are defined in the literature (38).

Previous studies addressed the topic of graph measures in schizophrenia (6, 39), and it is not the purpose of the current study to repeat these assessments. However, the purpose of analyzing graph modularity in these data is to compare with the other two measures (connectivity strength and randomness) included in this work. A similar comparison has been previously performed in a different data set as it may be helpful in interpreting randomness results for domain connectivity (10). In the current case, we compare randomness and modularity for whole brain FNC only. Modularity values (denoted as *Q*) were calculated using the Newman’s method (40) included in the Brain Connectivity Toolbox (https://sites.google.com/site/bctnet/Home) (38). Before statistical analysis, we checked for normality and applied the square root of *Q* to pass the Lilliefors normality test (41). The Lilliefors method tests the null hypothesis that data come from a normally distributed population. This test is useful when the true parameters of the distribution (mean and variance) are unknown and must be estimated from the given sample data. We repeated all linear regression and group analysis tests used for whole brain connectivity strength, but applied to modularity.

### Randomness Analysis

Randomness analysis of FNC matrices is based on random matrix theory (10). The first difference with graph theory is that the matrix array is not assumed to describe a graph or an adjacency matrix. For example, one of the first applications of random matrices was the description of a larger atomic nucleus (42). Just as in connectivity strength, the randomness analysis is not based in graph theory and the reader should not try to make a strict connection with graph analysis. Randomness and graph theory may overlap in the case that the matrix of interest can be used to estimate an adjacency matrix. In the case of whole brain analysis, a transformation from FNC to adjacency is possible; thus, a comparison between randomness, connectivity strength, and modularity can be made. Such comparison helps provide an interpretative baseline given the existence of previous schizophrenia results on the modular organization of the brain (39).

A small randomness value *L* indicates that numbers in a connectivity matrix are normally distributed. Besides its known statistical properties, randomly drawn numbers do not exhibit a particular structure. Thus, non-significant *L* values are an indication of a connectivity matrix with little structure which coincides with a low modularity *Q* value. Significant *L* values are an indication of non-random structures in the connectivity matrix because it is unlikely that structure happens by chance. It is reasonable for values *L* and *Q* to be correlated in spite of coming from different theoretical frameworks. The most valuable advantage of randomness is that it can be applied to a rectangular matrix (matrices with different numbers of rows and columns) which is a common characteristic of domain connectivity matrices. More information about the randomness measure can be found in the supplement provided.

We estimated randomness for each subject, but given that *L* might have a skewed histogram; we used two transformations *T*{*L*} (an inverse square for the whole brain matrix and a fourth root for the domain submatrices) to increase Gaussianity and tested normality using the Lilliefors test (41). We examined randomness effects using the same whole brain and domain level analysis employed in the Connectivity Strength subsection, except that *T*{*L*} (the transformed randomness measure) was used as the dependent variable instead of connectivity strength.

## Results

### Whole Brain Analysis

**Figure 2:**
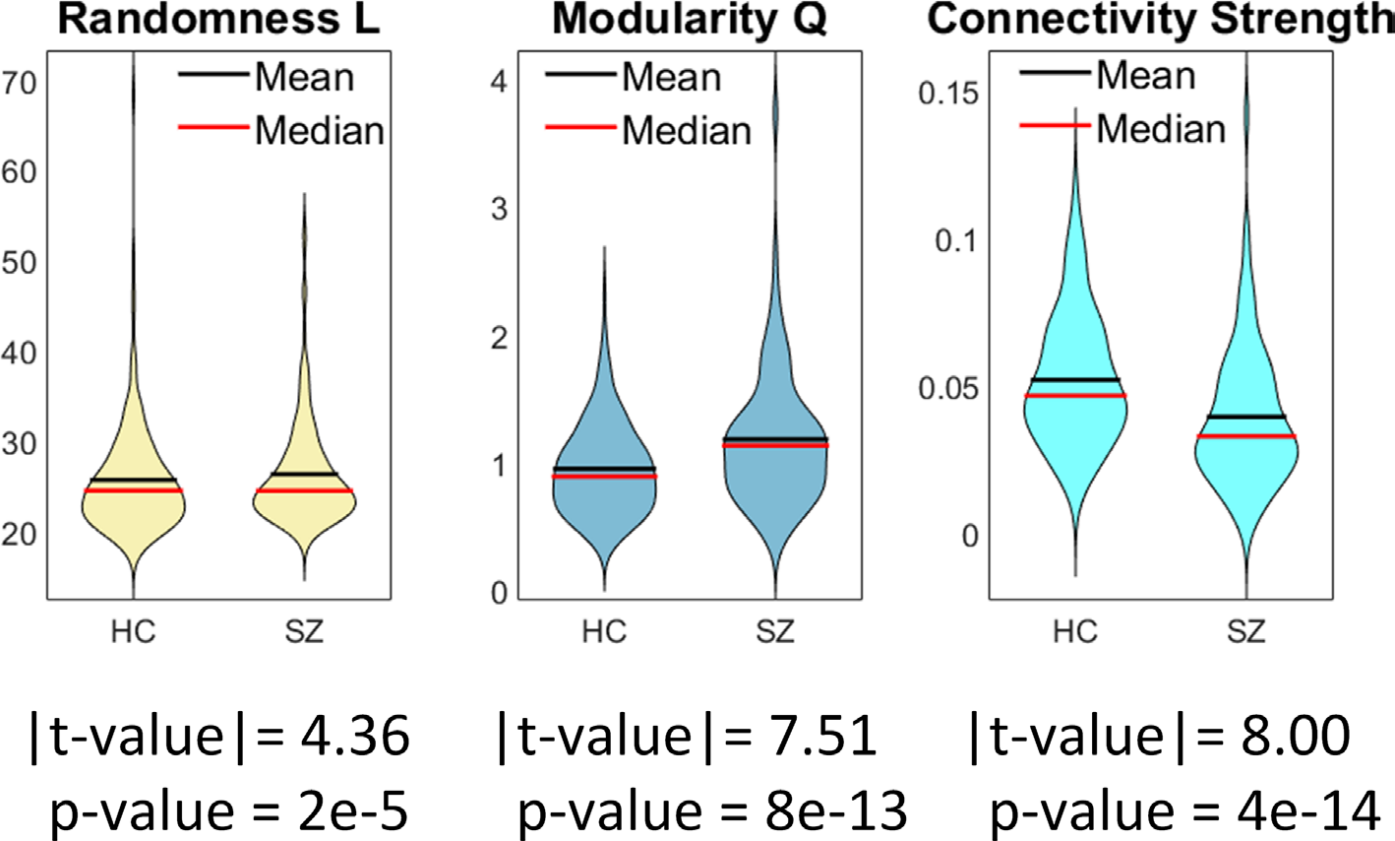
Whole brain group comparisons for connectivity strength, randomness (*L* value), and graph modularity (*Q* value). This figure displays the mean absolute measures (no transformations). Statistical tests were performed on normalized data after Gaussian distribution of transformed data was verified using Lilliefors tests.

Compared to healthy volunteers, individuals with schizophrenia had less random connectivity (higher randomness value *L*), higher modularity, and lower connectivity strength (see **Figure 2**). Group differences in randomness and modularity were significant with *p* values below 2e−5 for the transformed values. The connectivity strength was significantly lower in individuals with schizophrenia compared to controls with a *p* value of the order of 10^−14^. Moreover, the composite CMINDS cognitive performance score exhibited positive relationships with whole brain connectivity strength (*p* < 0.047). In addition, composite and visual learning CMINDS scores exhibited negative relationship with Modularity *Q* (*p* < 0.02). Since the CMINDS scores could have been influenced by diagnosis, we further tested a regression model including the interaction of diagnosis with CMINDS which showed no significant interaction terms. No CMINDS score was significantly related to randomness. There was not significant result in any whole brain analyses related to PANSS. Details of these results are shown in **Supplementary Table 2**.

**Figure 3:**
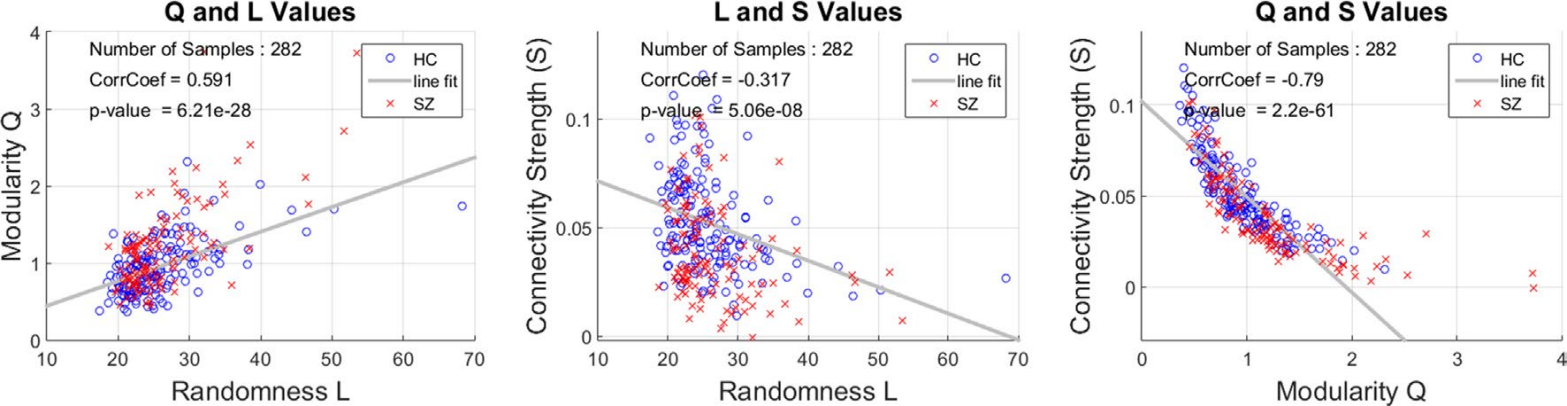
Correlations among connectivity strength *S*, randomness *L*, and modularity *Q*. The correlation between *L* and *Q* is strong (*r* = 0.591). Each point corresponds to a single subject’s whole functional network connectivity (FNC) matrix. *L* and *Q* are negatively correlated with the connectivity strength measure *S*.

The group differences in randomness (*L*) and modularity (*Q*) exhibited the same direction of effect, similar to prior observations that these two measures were moderately correlated based on the use of a binary graph of *Q* (10, 43). An important difference in the current analysis is the use of a weighted rather than a binary graph. This motivated us to further analyze the relationship between connectivity strength (*S*), *L*, and *Q*. This relationship is important when interpreting per-domain analyses. The correlation between *L* and *Q* (**Figure 3**) based on the use of a weighted graph is high (0.59), which is similar to the previously reported binary graph assessment (10). The correlations *L* vs. *S* (−0.32) and *Q* vs. *S* (−0.79) were both significantly negative (*p* < 4e−6). Results from group testing and correlations among the three measures are consistent on their direction of effect.

### Domain Analysis

**Figure 4:**
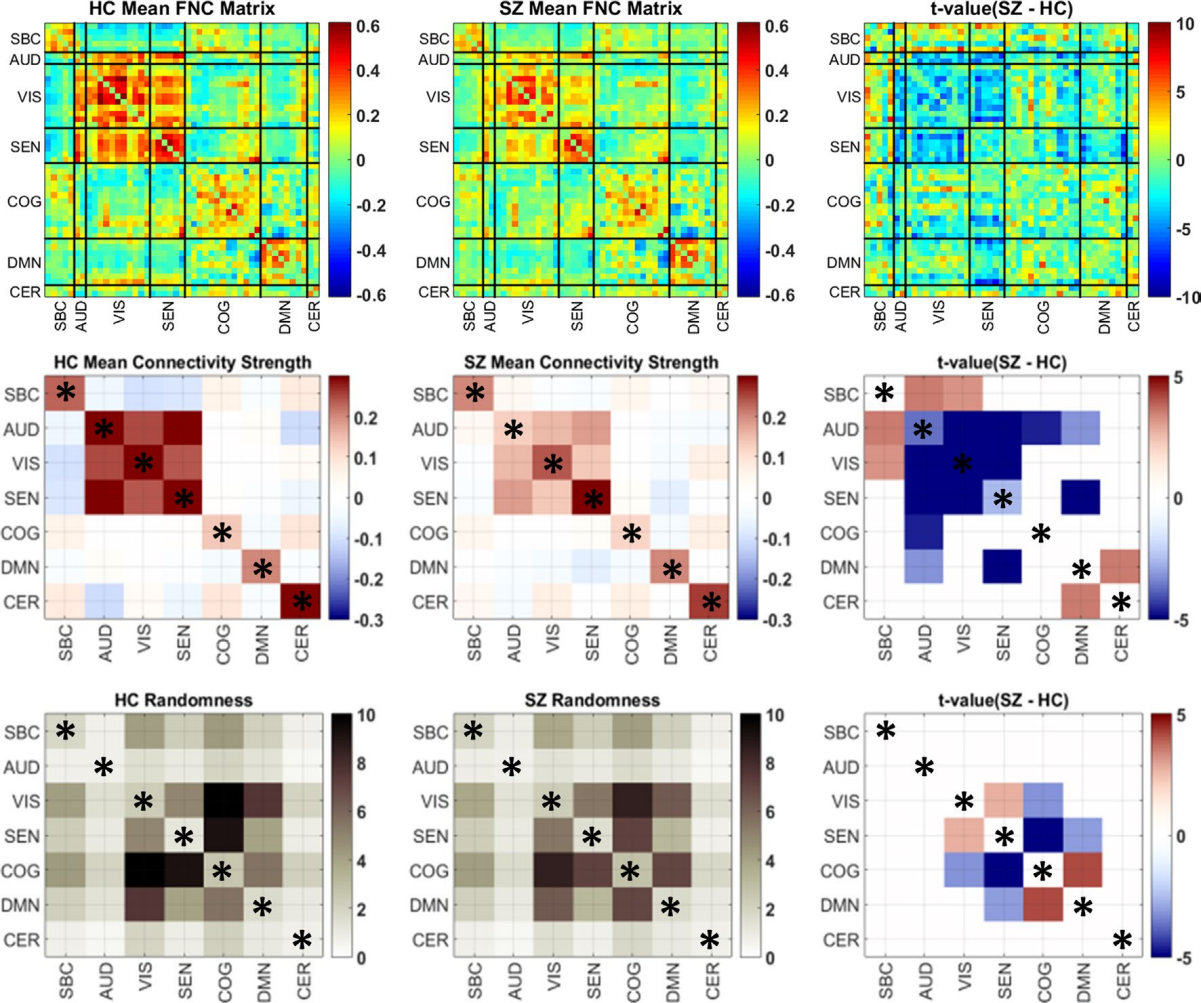
Group mean and differences in connectivity strength and randomness matrices. The first row displays the original FNC matrices for each group and the *t* values comparing the two groups. Domain submatrices are delimited by black lines. Because domain analysis estimates one single value per submatrix, it is easier to illustrate each submatrix value using squares of the same size. Thus, submatrix size is ignored in the second and third rows only for illustration purposes. The number of submatrix elements for significant results are included in **Tables 1** and **2**. The last column portrays only significant *t* values after Gaussianity transformation (Lilliefors test) and false discovery rate (FDR) multiple comparison correction. Domains have been named as sub-cortical (SBC), auditory (AUD), visual (VIS), sensorimotor (SEN), cognitive control (COG), default-mode network (DMN), and cerebellum (CER). Within domain results are marked by an asterisk.

The partitioning of the whole brain matrix into domain submatrices can be found in **Figure 1**. Domain analysis was independently performed on each of the submatrices displayed in the figure. There were significant group differences in randomness (*L*) and connectivity strength among domain submatrices (**Figure 4**). Compared to healthy controls, connectivity strengths were lower in schizophrenia within and between all AVSN (audio-visual and sensorimotor) domains (**Figure 4** and **Table 1**). Similarly, between domain connectivity strength was lower in schizophrenia for COG-AUD, SEN-DMN, and AUD-DMN. In contrast, schizophrenia subjects exhibit higher connectivity strength in the case of SBC-AUD, SBC-VIS (sensorial input and subcortical), and DMN-CER domains.

**Table 1:** Significant group differences in connectivity strength for within and between domain assessments. Submatrices of each domain pair are displayed in Figure 1.

Type (# submatrix elements)	Number of singular values	Domain 1	Domain 2	HC mean *S*	SZ mean *S*	Cohen’s *D*	*t* value	*p* value
**SZ > HC**								
Between (10)	2	SBC	AUD	−0.05	0.04	0.42	3.48	5.78E−04
Between (55)	5	SBC	VIS	−0.10	−0.02	0.38	3.13	1.94E−03
Between (16)	2	DMN	CER	−0.02	−0.01	0.44	3.66	3.03E−04
**SZ < HC**								
Within (1)	2	AUD	AUD	0.30	0.11	−0.44	−3.65	3.12E−04
Between (22)	2	AUD	VIS	0.25	0.15	−0.78	−6.51	3.48E−10
Between (12)	2	AUD	SEN	0.31	0.17	−0.74	−6.16	2.52E−09
Between (26)	2	AUD	COG	0.01	0.001	−0.54	−4.50	9.76E−06
Between (16)	2	AUD	DMN	0.02	−0.02	−0.38	−3.15	1.83E−03
Within (55)	11	VIS	VIS	0.33	0.23	−0.62	−5.17	4.57E−07
Between (66)	6	VIS	SEN	0.24	0.13	−0.71	−5.88	1.20E−08
Within (15)	6	SEN	SEN	0.40	0.31	−0.34	−2.80	5.50E−03
Between (48)	6	SEN	DMN	−0.02	−0.07	−0.78	−6.47	4.30E−10

**Figure 5:**
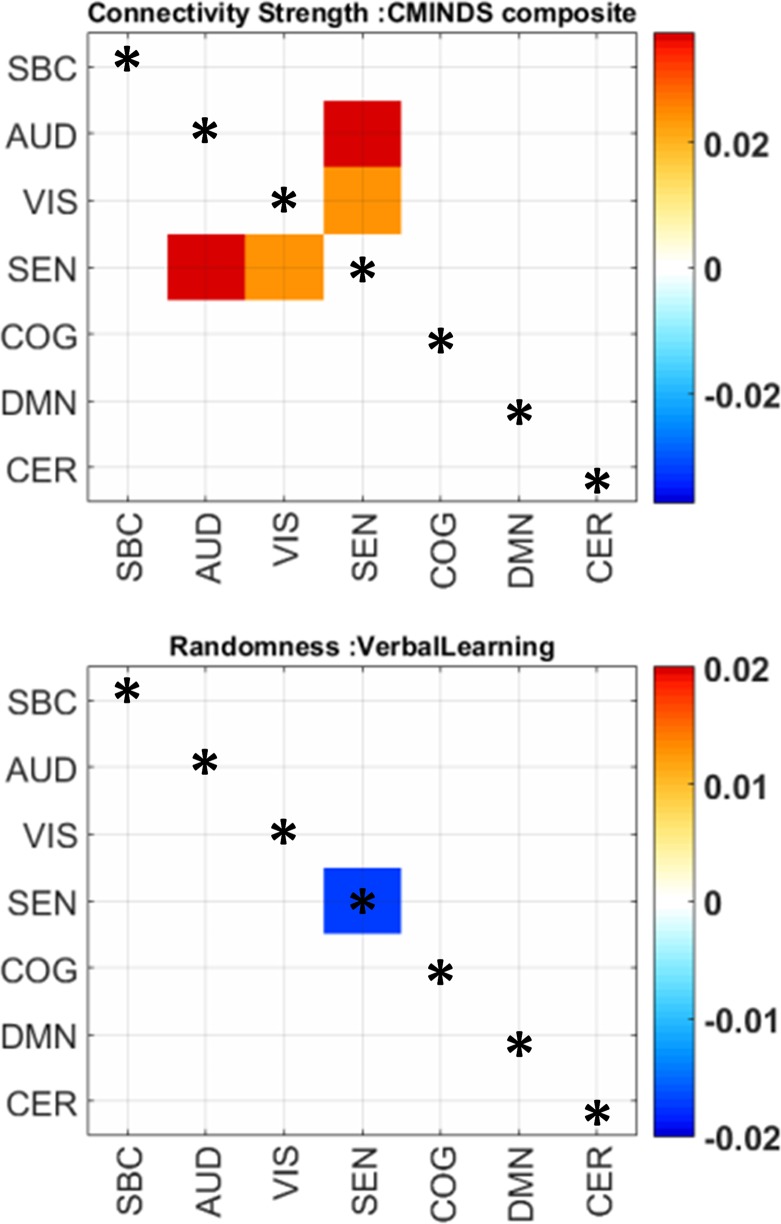
Significant relationships between randomness, connectivity strength, and CMINDS scores. The color scale indicates beta values. Only significant regression coefficients are displayed; the non-significant cells are white. Within domain results are marked by an asterisk.

While group differences of domain connectivity strength were prominent within sensory processing areas, randomness did not exhibit many differences within AVSN domains. Instead, there are three significant differences in COG-VIS, COG-SEN, and DMN-SEN with more random submatrices (lower *L*) in SZ subjects. In addition, between connectivity in DMN-COG and SEN-VIS were less random in SZ (higher *L*) than HC subjects. These results are displayed in **Figure 4** and **Table 2**. As described in previous work, a lower value *L* (more randomness) decreases the chance of finding structure in the domain connectivity matrices (10). This last statement relates to the correlation between modularity and randomness in **Figure 3** since larger *L* (less randomness) correlates with larger *Q* (higher modularity structure). The analysis of CMINDS effects showed three significant results. Connectivity strength was positively associated with the CMINDS Composite score in SEN-VIS and SEN-AUD between connectivity domains. Randomness value *L* was negatively associated with the CMINDS Verbal Learning score in the within connectivity SEN-SEN. These results are displayed in **Figure 5**. There was one significantly negative relationship (*p* < 0.0011) that passes FDR correction between Randomness value *L* and the interaction term Diagnosis X Reasoning Problem Solving CMINDS score for the between connectivity of SBC-VIS domains. A complete statistical report can be found in **Supplementary Table 3** and **Supplementary Table 4**.

**Table 2:** Significant group differences of randomness for within and between domain assessments. Submatrices of each domain pair are displayed in **Figure 1**.

Type (# submatrix elements)	Number of singular values	Domain 1	Domain 2	HC mean *L*	SZ mean *L*	Cohen’s *D*	*t* value	*p* value
**SZ > HC**								
Between (66)	6	VIS	SEN	4.95	5.41	0.33	2.74	6.51E−03
Between (104)	8	COG	DMN	5.47	7.13	0.50	4.15	4.36E−05
**SZ < HC**								
Between (143)	11	VIS	COG	10.53	8.37	−0.38	−3.13	1.94E−03
Between (78)	6	SEN	COG	9.19	6.96	−0.67	−5.52	7.87E−08
Between (48)	6	SEN	DMN	4.00	2.99	−0.37	−3.05	2.54E−03

## Discussion

The current work looks for functional connectivity abnormalities related to schizophrenia at whole brain and domain (groups of RSNs) levels. Previous studies of resting state functional connectivity have found many differences between schizophrenia patients and healthy subjects at more granular brain segmentations based on RSNs (5). Our results show that granularly localized abnormalities affect connectivity at coarser spatial levels. At the coarsest level, findings show that average whole brain connectivity is lower and modular structure is larger in schizophrenia patients. The enhanced connectivity structure in schizophrenia is confirmed by both randomness and graph modularity measures. Several connectivity results at the domain level were similar to those observed at the RSN level specifically the similarity in connectivity effects of SBC and AVSN domains (5). However, some observed abnormalities were not reported in the previous RSN analysis: 1) higher connectivity between cerebellum and DMN; 2) lower connectivity between cognitive and auditory domains; 3) lower connectivity between DMN and sensory input domains (AUD and SEN). In addition, schizophrenia subjects exhibit less randomness for the between connectivity DMN-COG and SEN-VIS; and more randomness for some cognitive-sensorial (COG-VIS and COG-SEN) and DMN-sensorimotor.

The current results are compatible with the previous ones where increments of functional connectivity in schizophrenia relative to healthy controls were found between SBC with AVSN domains and decrements were found within sensory input and motor domains. It is no surprise that Cohen’s *D* showed medium effect sizes among SBC and AVSN results in **Table 1**. However, the results in **Figure 4** show an extra set of dysfunctions that were likely found due to the averaging of correlations allowing for a higher detection power. We can see higher cerebellum-DMN connectivity in schizophrenia. Results with the cerebellum-DMN exhibited a similar effect size (low to medium Cohen’s *D*) as that found in the subcortical connectivity. While previous RSN-based analysis did not report substantial number of differences within these domains (5), domain analysis showed there are additional connectivity effects in DMN, cerebellum, cognitive control, and attention domains related to schizophrenia.

Previous work determined the existence of dysfunctional connectivity between thalamus and AVSN RSNs (5). These results are consistent with observations in the literature (44), and it is possible that the thalamus was the major contributor to the subcortical results observed in this work. Notice that the subcortical domain includes putamen and caudate in addition to the thalamus since we are analyzing the whole subcortical domain as a collection of several RSNs. Thus, detected effects are not restricted to the thalamus alone but are contextualized to the domain as a group of RSNs. An important new finding is the lower connectivity between DMN-AUD and DMN-SEN domains in schizophrenia compared to control subjects. Effects in aggregated connectivity differ from those of pinpointed brain areas. This might be the case of the cerebellum area where reports in the literature suggest that some cerebellum areas exhibit a decreased connectivity with cortical areas (45). However, effects of connectivity strength in our results indicate a significant connectivity increment between cerebellum and DMN areas. These discrepancies likely characterize the difference between testing for one single connectivity measure and using the aggregation of connectivity values from a larger collection of brain areas.

Random connectivity patterns are a different metric compared to correlation-based measures of relationship between two domains. As **Figure 3** indicates, graph modularity and randomness are highly correlated indicating that randomness and modularity share common characteristics. However, both concepts address different aspects that can be measured from a connectivity matrix (43). In the whole brain adjacency matrix case, it was possible to assess this similarity between randomness and modularity which showed consistency when comparing against connectivity strength. In our data, as connectivity strength decreases, both randomness and modularity measures increase. The SEN-VIS result is another outcome where lower domain connectivity was concurrently observed with less randomness. A full mathematical analysis of this effect is not available at the moment and might be a topic of future study. While the inverse relationship between connectivity strength and graph modularity/randomness could appear contradictory, the following example illustrates why this is not the case. For example, take a fully connected graph where the connectivity matrix is full of ones; thus, the average connectivity is 1 but it has a low modularity equal to zero because there is only one module. Consider now a connectivity matrix with a chessboard pattern of zeros and ones where the average connectivity is 0.5 (equal number of zeros and ones) but now the modularity measure is equal to 0.5. This example has been explored using modularity and randomness (10) and is useful illustrating that lower connectivity might result in higher graph structure which reflects in randomness and modularity measures. The inverse relationships observed in **Figure 3** are then reasonable. In summary, a larger value *L* (lower randomness) in schizophrenia is similar to a larger modularity (larger *Q* value) indicating more structure in the connectivity data and a lower probability that such structure occurred by chance. In the case of domain analysis, randomness allowed the observation of changes in connectivity structure that cannot be measured using graph measures. As explained in the Methods section, domain connectivity matrices have properties that do not permit the estimation of an adjacency matrix. To interpret the results, we can mention that increments in the randomness measure *L* are associated with a less random submatrix structure. Since it has been shown in **Figure 3** that randomness and modularity are correlated, it is possible to argue that randomness allows for the assessment of structure within domain connectivity in spite of not being able to employ modularity. However, we must keep in mind that modularity is a measure of community structure for graphs (40), but randomness is not assessing the existence of these communities. Nevertheless, it is reasonable to assume that existence of community structure correlates with decreased randomness.

The main outcomes of randomness analysis were centered on the DMN, COG, VIS, and SEN domains. The first result worth mentioning is a less random relationship between COG and DMN domains. Based on this result, we can argue that connectivity between these two domains tends to have a less random structure in SZ patients. Since connectivity strength was not significantly different in the COG-DMN domains, we can argue that the main dysfunction between COG and DMN areas is related to its connectivity pattern instead of its strength. This outcome is in line with the hypothesis that dysfunctional DMN could intrude in cognitive functioning of the brain (46). Since the group test shows less randomness in COG-DMN for SZ patients, this result could indicate a dysfunctional restructuring of connectivity affecting cognition in schizophrenia. This structuring could also be a compensation trade-off between the decrement of randomness in COG-DMN against the increment of randomness between VIS-COG, SEN-DMN, and SEN-COG domains. The next outcome to mention is the decreased randomness in SEN-VIS domains accompanied by reduced connectivity strength. In this case we argue that connectivity decrements are different for each RSN pair resulting in an uneven reduction effect through the SEN-VIS submatrix. As observed in **Figure 4**, the DMN seems to be more sensitive to dysfunctions related to schizophrenia since it suffered lower connectivity strength with AUD and SEN domains, as well as altered randomness behavior with respect to the connectivity with SEN and COG domains.

Findings in this study were possible because the focus was domain connectivity, which is not too coarse as whole brain analysis or as fine as single RSN analysis which might be spatially constrained. This focus on a relatively intermediate spatial resolution has the strength of revealing abnormalities of functionally related brain areas that might not be strong unless analyzed as a group. Observations in this study were consistent with previous per-RSN analysis (5) confirming that strong effects at a finer RSN resolution effectively translates into effects of functionally grouped RSNs. Furthermore, the main advantage of domain analysis was its sensitivity to domain effects that were not previously observed. The main limitation of the method is the difficulty in identifying a specific brain area for effects, since results pertain to grouped RSNs. This limitation is a trade-off for detection power since the aggregation of several connectivities allowed observing effects not seen for individual RSNs. This limitation can be overcome simply by turning to consider individual functional connectivities. For example, studying individual connectivities allowed identifying the thalamus as the subcortical area with stronger and significant effect (5) albeit missing many of the domain effects reported here. Applying modularity was another limitation when analyzing domain connectivity. In this work, we tried overcoming this limitation indirectly by using randomness as a measure highly correlated to modularity. Another important limitation is the short fMRI scanning time of 5 min. There is current controversy through the literature whether this is too short or appropriate (47, 48). There is a recent warning of increased probability of entering sleepiness near 7 min (49, 50). Resting state experiments have been criticized regarding the existence of these sleep states (51). However, our data have a large probability of avoiding contamination by sleep states staying within 5 min. Another limitation in our data is the spatial resolution set by the 4 mm slice thickness of the fMRI scanning protocol. It is likely that signals from small brain structures were not resolved. A new pattern of aberrant dysfunctions was observed from the randomness analysis. These effects would not have been possible to be measured using modularity only because of its mathematical restrictions with respect to the connectivity matrices involved.

## Conclusion

Analyzing functional connectivity using a domain framework confirmed several effects observed in schizophrenia and identified previously unobserved effects. Whole brain connectivity was lower in individuals with schizophrenia compared to controls suggesting deleterious overall effects. Whole brain results showed less randomness in SZ, but this change in the overall connectivity might be a detrimental characteristic in the brain of SZ subjects. Dysfunctional connectivity between subcortical and sensory areas is consistent with reports from the literature. In contrast, we found lower connectivity between cognitive-auditory, DMN-auditory, and DMN-sensorial brain areas. With respect to randomness there are two distinct effects. Connectivity patterns between DMN and COG domains appear less random, while patterns among DMN-sensorial and COG-sensorial areas appear more random in individuals with schizophrenia compared to controls. These findings may suggest a compensation mechanism that promotes more structure (less random) in connectivity patterns within the higher cognitive function domains due to a deleterious relationship among areas processing external signals. At the same time, dysfunctional DMN relationship with COG has been seen as pernicious (46).

## Ethics Statement

All subjects gave written informed consent to participate and the study was approved by the Institutional Review Boards of the following participating data collection sites included in this work: University of California Irvine, the University of California Los Angeles, the University of California San Francisco, Duke University, University of North Carolina, University of New Mexico, University of Iowa, and University of Minnesota.

## Author Contributions

VV and VC contributed to method design and implementation of the study. ED performed data analysis. JT, GP, AB, DM, SP, AP, JV, SM, and TE conducted data acquisition. All authors contributed to the manuscript revision, read and approved the submitted version.

## Funding

This work was supported by grants from the National Institutes of Health grant numbers 2R01EB005846, R01REB020407, and P20GM103472; and the National Science Foundation (NSF) grants 1539067/1631819 to VDC.

## Conflict of Interest Statement

The authors declare that the research was conducted in the absence of any commercial or financial relationships that could be construed as a potential conflict of interest.
