# Supplementary figures and images for: Altered Domain Functional Network Connectivity Strength and Randomness in Schizophrenia
Source: Front Psychiatry. 2019 Jul 23;10:499. doi: 10.3389/fpsyt.2019.00499 (PMC6664085; doi:10.3389/fpsyt.2019.00499)

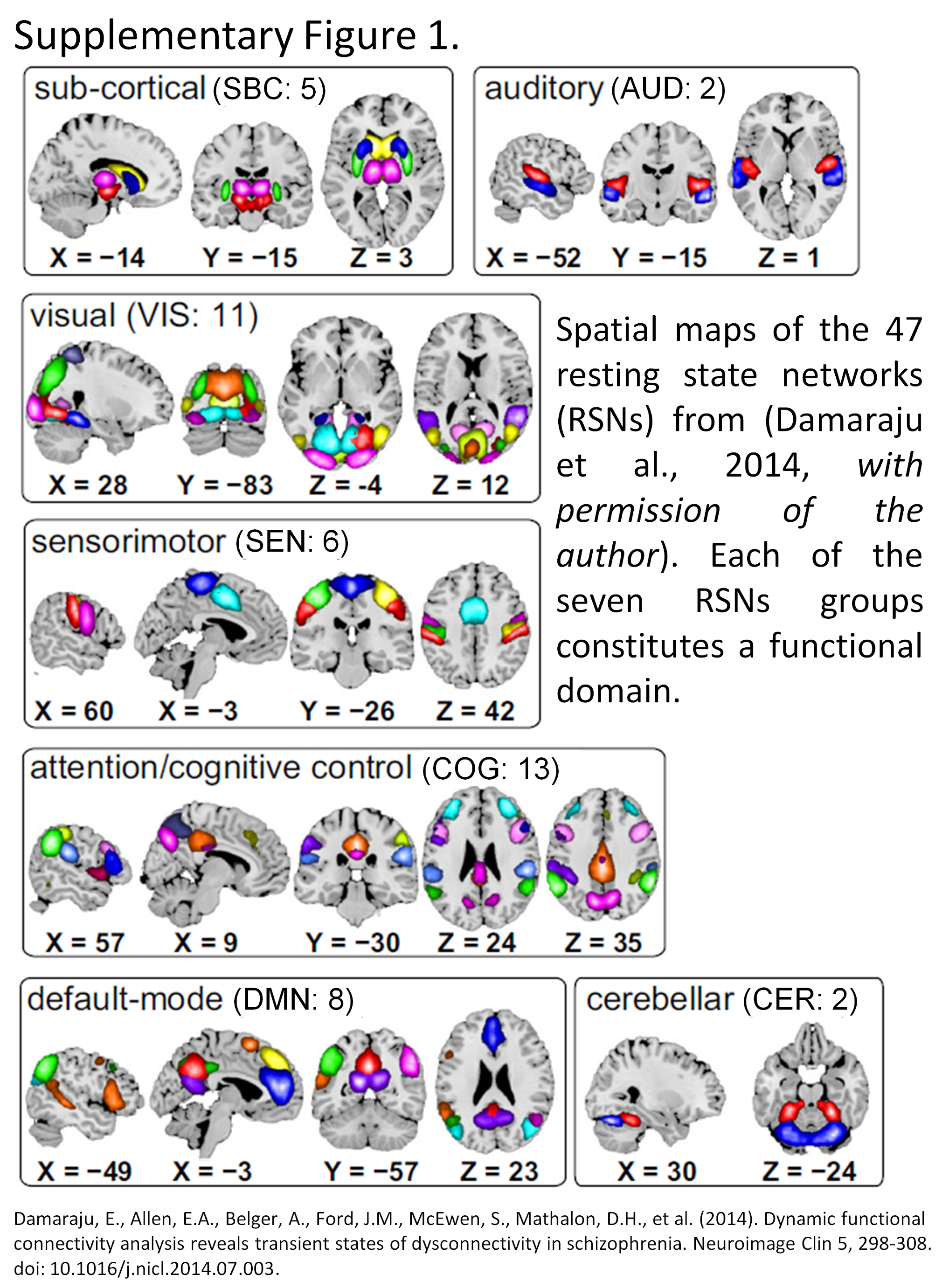

Supplement: Supplementary file 1 [file Image_1.tif]
