# Supplementary material for: Altered Domain Functional Network Connectivity Strength and Randomness in Schizophrenia
Source: Front Psychiatry. 2019 Jul 23;10:499. doi: 10.3389/fpsyt.2019.00499 (PMC6664085; doi:10.3389/fpsyt.2019.00499)
Supplement: Supplementary file 6 [file DataSheet_1.docx]

Supplementary Material

Altered Domain Functional Network Connectivity Strength and Randomness in Schizophrenia

Victor M. Vergara, Eswar Damaraju, Jessica A. Turner, Godfrey Pearlson, Aysenil Belger, Daniel Mathalon, S.G. Potkin, A. Preda, J.G. Vaidya, T.G.M. van Erp, S.McEwen, and Vince D Calhoun

This supplement provides a more detailed description of the methodology used in this manuscript.

## Connectivity Strength

The functional network connectivity (FNC) approach employed estimates temporal variations of brain activity, which we refer to time courses, and where in the brain this activity occurs by means of a group independent component analysis (gICA) (Calhoun and Adali, 2004). Connectivity estimation is performed using the Pearson correlation between two different time courses. This is illustrated in Figure 1 for the two time courses X(t) and Y(t). A table with correlations is necessary after gICA since there are more than just two brain areas involved. The table with correlation represents a matrix since it is a numeric array of numbers. Numbers in the correlation matrix are transformed into colors because to avoid problems with fonts in illustrations. This procedure is illustrated in Figure 1.


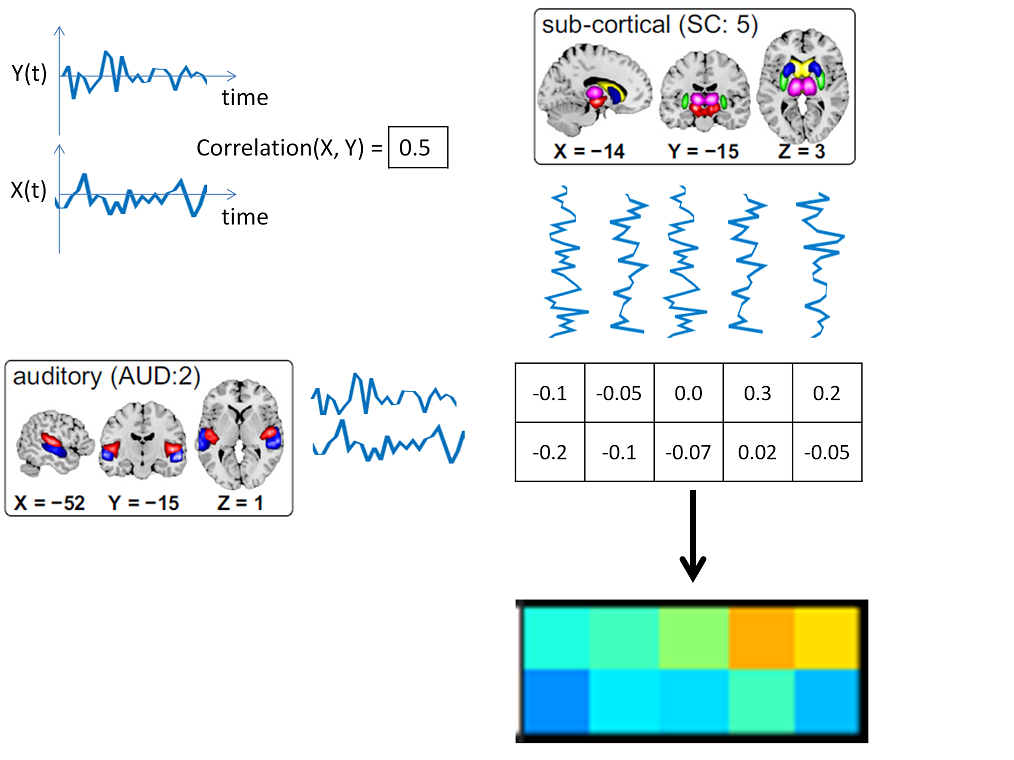


Figure 1. Correlation is calculated from time courses describing brain activation changes with time. Since more than one correlation is obtained, the values of domains involved are positioned in a table with all correlations. The table is a correlation matrix which is transformed to a color map for illustration purposes.

The connectivity strength used in this work is estimated from the connectivity matrix simply by averaging all numbers in the matrix. Notice that averaging does not consider the position of the numbers in the matrix. The resulting average number is then represented as a color in the colormap as seen in Figure 2.


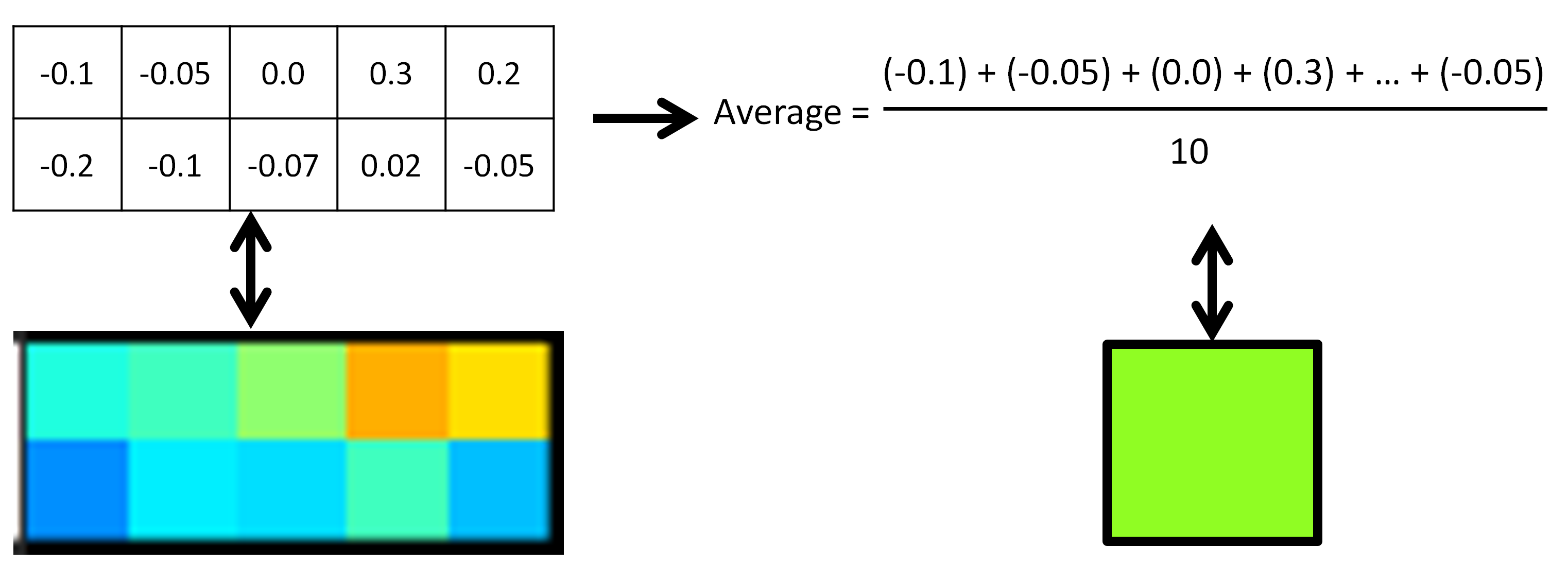


Figure 2. Connectivity strength is obtained by averaging all tabulated correlations.

## Symmetric Matrices in Within Connectivity

Different from the simple average used in connectivity strength, more complete assessments of connectivity employs rely on the structure of the matrix. Figures 1 and 2 show a two by five table/matrix where the connectivity is estimated **between** domains auditory (AUD) and sub-cortical (SBC) areas. Most of the connectivity matrices are of this between domain type. In contrast, Figure 3 shows a connectivity matrix **within** the SBC domain with different properties not observed in Figure 2. One is caller rectangularity since the matrix has the same number of rows and columns. The second is symmetry along the main diagonal of the matrix. These two properties are key factors of the other measures used in this manuscript.


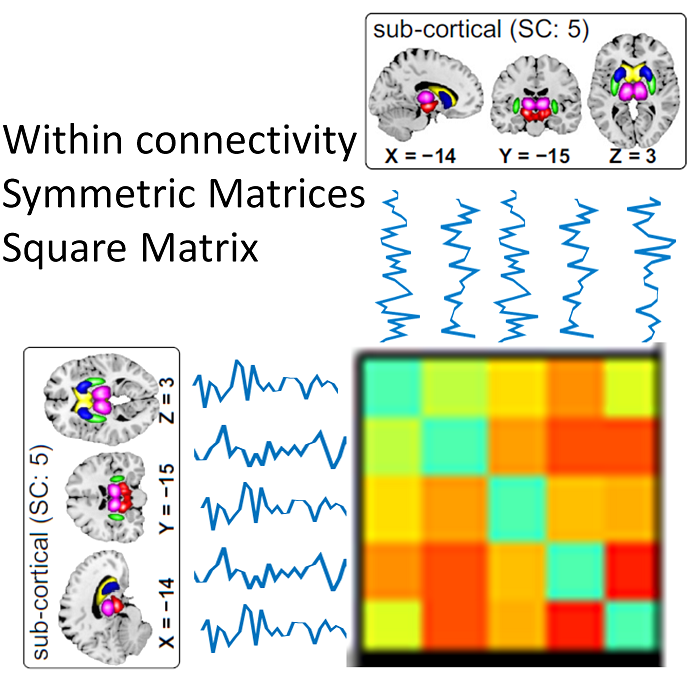


Figure 3. Tabulating the within domain connectivity results in a symmetric matrix. The symmetry can be visually seen along the diagonal from the upper left corner to the lower right corner.

# Graph Modularity

In graph modularity we construct a connectivity matrix similar the same way it was done in the first section of this supplement. The easiest example is to consider connected or disconnected relationships among brain areas. Figure 4 shows an example for a very small graph of four nodes created for illustration. Modularity is a measure of the community structure of a graph. Each community may be a set of interconnected nodes separated from other sets of nodes. When all nodes are connected to each other there is no structure and all nodes belong to a single community resulting in the smallest modularity (Q = 0). Figure 4 also shows an example with two communities of two nodes each one. In that case the modularity value increases = 0.5. Different from the average connectivity strength, the position of the ones and zeros in the matrix is crucial for the estimation of modularity. A complete mathematical description of the modularity assessment can be found in {Newman, 2006 #406}. This method assumes knowledge of the connection status of all nodes. However, this is not possible in the connectivity matrix of Figure 1 because only connections between AUD nodes and SBC nodes are known, but within connectivity SBC to SBC and AUD to AUD nodes are unknown. This simple example shows how between domains connectivity has an undefined modularity measure as defined graph theory metrics.


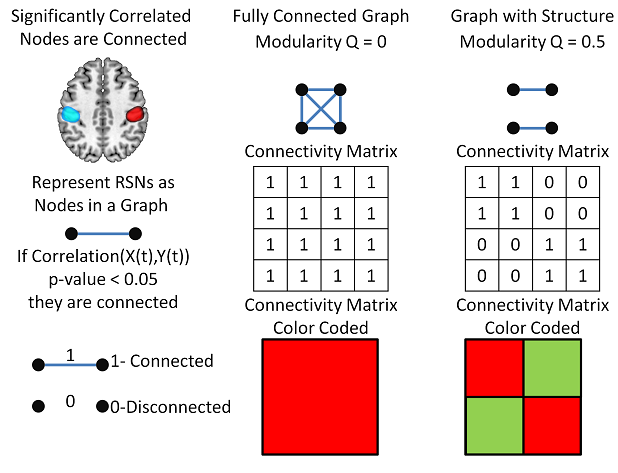


Figure 4. A criterion for assessing connectivity between brain areas is necessary. This figure shows an easy criterion for illustration purposes. Significant correlations (p < 0.05) represent a connection denoted as 1, otherwise disconnection denoted as 0. Fully connected graphs have no modularity thus Q = 0. Partially connected graphs may exhibit community structure. In this example the whole graph with four nodes was connected to exhibit two different small communities of two nodes each. The modularity is then Q = 0.5.

## Randomness Analysis

The randomness assessment aims at determining if the correlation values in the connectivity table/matrix were selected from a normal distribution or not. The randomness L measure is small and approaches zero as the correlations in the connectivity table/matrix are similar to a set of numbers drawn from a Normal distribution. For example, it is very unlikely that a matrix with structure like the one in figures 4 and 5 happens just by chance. In this case, the L value is large. The randomness measure L has a closed form p-value equation that can help to determine if the matrix is random or not. A complete mathematical description is presented in (Vergara et al., 2018). The randomness method compares the singular values (SVs) $\lambda_{i}$ of an FNC matrix against the mean SVs $\mu_{i}$ of random matrices utilizing a Mahalanobis distance. The Mahalanobis distance requires estimation of SVs $\mu_{i}$ and the covariance matrix $\boldsymbol{\Sigma}$ of SVs from random matrices. The estimation is obtained after considering a large number of random matrices with matrix elements drawn from the same Gaussian distribution. Considering vectors $\boldsymbol{\lambda}$ (with all $\lambda_{i}$ from the FNC matrix of interest) and $\boldsymbol{\mu}$ (with all $\mu_{i}$ from random matrices) the randomness measure is written as $L=\left( \boldsymbol{\lambda-\mu} \right)\boldsymbol{\Sigma}\left( \boldsymbol{\lambda-\mu} \right)^{T}\mathbf{/}N$, where $N$ is the number of SVs. The value $NL$ follows a chi square distribution employed to test the null hypothesis that all elements in the FNC matrix comes from the same Gaussian distribution (Vergara et al., 2018).


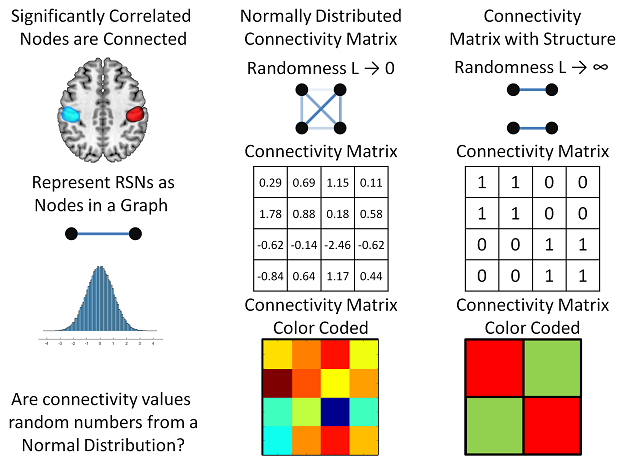


Figure 5. The randomness measure aims at determining of connectivity matrix is just a set of random numbers drawn from a Normal distribution. If numbers follow a Normal distribution then L is very small and tends to zero. Otherwise, the L measure is very large.

# References

Anticevic, A., Cole, M.W., Repovs, G., Murray, J.D., Brumbaugh, M.S., Winkler, A.M., et al. (2014). Characterizing thalamo-cortical disturbances in schizophrenia and bipolar illness. *Cereb Cortex* 24(12)**,** 3116-3130. doi: 10.1093/cercor/bht165.

Bastian, A.J. (2006). Learning to predict the future: the cerebellum adapts feedforward movement control. *Curr Opin Neurobiol* 16(6)**,** 645-649. doi: 10.1016/j.conb.2006.08.016.

Birn, R.M., Molloy, E.K., Patriat, R., Parker, T., Meier, T.B., Kirk, G.R., et al. (2013). The effect of scan length on the reliability of resting-state fMRI connectivity estimates. *Neuroimage* 83**,** 550-558. doi: 10.1016/j.neuroimage.2013.05.099.

Calhoun, V., and Adali, T. (2004). Group ICA of fMRI toolbox (GIFT). *Online at* [*http://icatb*](http://icatb)*. sourceforge. net*.

Damaraju, E., Allen, E.A., Belger, A., Ford, J.M., McEwen, S., Mathalon, D.H., et al. (2014). Dynamic functional connectivity analysis reveals transient states of dysconnectivity in schizophrenia. *Neuroimage Clin* 5**,** 298-308. doi: 10.1016/j.nicl.2014.07.003.

Danckert, J., and Merrifield, C. (2016). Boredom, sustained attention and the default mode network. *Exp Brain Res*. doi: 10.1007/s00221-016-4617-5.

Desmond, J.E., and Fiez, J.A. (1998). Neuroimaging studies of the cerebellum: language, learning and memory. *Trends in Cognitive Sciences* 2(9)**,** 355-362. doi: 10.1016/s1364-6613(98)01211-x.

Fornito, A., Yoon, J., Zalesky, A., Bullmore, E.T., and Carter, C.S. (2011). General and specific functional connectivity disturbances in first-episode schizophrenia during cognitive control performance. *Biol Psychiatry* 70(1)**,** 64-72. doi: 10.1016/j.biopsych.2011.02.019.

Gonzalez-Castillo, J., and Bandettini, P.A. (2017). Task-based dynamic functional connectivity: Recent findings and open questions. *Neuroimage*. doi: 10.1016/j.neuroimage.2017.08.006.

Gottwald, B., Mihajlovic, Z., Wilde, B., and Mehdorn, H.M. (2003). Does the cerebellum contribute to specific aspects of attention? *Neuropsychologia* 41(11)**,** 1452-1460. doi: 10.1016/s0028-3932(03)00090-3.

Gui, D., Xu, S., Zhu, S., Fang, Z., Spaeth, A.M., Xin, Y., et al. (2015). Resting spontaneous activity in the default mode network predicts performance decline during prolonged attention workload. *Neuroimage* 120**,** 323-330. doi: 10.1016/j.neuroimage.2015.07.030.

Haimovici, A., Tagliazucchi, E., Balenzuela, P., and Laufs, H. (2017). On wakefulness fluctuations as a source of BOLD functional connectivity dynamics. *Sci Rep* 7(1)**,** 5908. doi: 10.1038/s41598-017-06389-4.

Laumann, T.O., Snyder, A.Z., Mitra, A., Gordon, E.M., Gratton, C., Adeyemo, B., et al. (2016). On the stability of bold fmri correlations. *Cerebral Cortex*.

Lilliefors, H.W. (1967). On the Kolmogorov-Smirnov Test for Normality with Mean and Variance Unknown. *Journal of the American Statistical Association* 62(318)**,** 399. doi: 10.1080/01621459.1967.10482916.

Newman, M.E. (2006). Modularity and community structure in networks. *Proc Natl Acad Sci U S A* 103(23)**,** 8577-8582. doi: 10.1073/pnas.0601602103.

Petrosini, L. (1998). The cerebellum in the spatial problem solving: a co-star or a guest star? *Progress in Neurobiology* 56(2)**,** 191-210. doi: 10.1016/s0301-0082(98)00036-7.

Ray, K.L., Lesh, T.A., Howell, A.M., Salo, T.P., Ragland, J.D., MacDonald, A.W., et al. (2017). Functional network changes and cognitive control in schizophrenia. *Neuroimage Clin* 15**,** 161-170. doi: 10.1016/j.nicl.2017.05.001.

Sonuga-Barke, E.J., and Castellanos, F.X. (2007). Spontaneous attentional fluctuations in impaired states and pathological conditions: a neurobiological hypothesis. *Neuroscience & Biobehavioral Reviews* 31(7)**,** 977-986.

Stein, J.F. (1986). Role of the cerebellum in the visual guidance of movement. *Nature* 323(6085)**,** 217-221. doi: 10.1038/323217a0.

Tagliazucchi, E., and Laufs, H. (2014). Decoding wakefulness levels from typical fMRI resting-state data reveals reliable drifts between wakefulness and sleep. *Neuron* 82(3)**,** 695-708. doi: 10.1016/j.neuron.2014.03.020.

Tomlinson, S.P., Davis, N.J., Morgan, H.M., and Bracewell, R.M. (2014). Cerebellar contributions to verbal working memory. *Cerebellum* 13(3)**,** 354-361. doi: 10.1007/s12311-013-0542-3.

Vergara, V.M., Yu, Q., and Calhoun, V.D. (2018). A method to assess randomness of functional connectivity matrices. *J Neurosci Methods* 303**,** 146-158. doi: 10.1016/j.jneumeth.2018.03.015.

Vergara, V.Y., Q;Calhoun VD (2018). "Graph Modularity and Randomness Measures", in: *IEEE Southwest Symposium on Image Analysis and Interpretation.* (Las Vegas, NV: IEEE (in press)).

Wigner, E.P. (1967). Random Matrices in Physics. *SIAM Review* 9(1)**,** 1-23. doi: 10.1137/1009001.

Woodward, N.D., Karbasforoushan, H., and Heckers, S. (2012). Thalamocortical dysconnectivity in schizophrenia. *Am J Psychiatry* 169(10)**,** 1092-1099. doi: 10.1176/appi.ajp.2012.12010056.

Yu, Q., Plis, S.M., Erhardt, E.B., Allen, E.A., Sui, J., Kiehl, K.A., et al. (2011). Modular Organization of Functional Network Connectivity in Healthy Controls and Patients with Schizophrenia during the Resting State. *Front Syst Neurosci* 5**,** 103. doi: 10.3389/fnsys.2011.00103.

Zhuo, C., Wang, C., Wang, L., Guo, X., Xu, Q., Liu, Y., et al. (2018). Altered resting-state functional connectivity of the cerebellum in schizophrenia. *Brain Imaging Behav* 12(2)**,** 383-389. doi: 10.1007/s11682-017-9704-0.
